# Identification of gene pairs through penalized regression subject to constraints

**DOI:** 10.1186/s12859-017-1872-9

**Published:** 2017-11-03

**Authors:** Rex Shen, Lan Luo, Hui Jiang

**Affiliations:** 1 The Blake School, Minneapolis, 55403 MN USA; 2 Department of Biostatistics, University of Michigan, Ann Arbor, 48109 MI USA

**Keywords:** Gene pair, Biomarker, Penalized regression, ADMM

## Abstract

**Background:**

This article concerns the identification of gene pairs or combinations of gene pairs associated with biological phenotype or clinical outcome, allowing for building predictive models that are not only robust to normalization but also easily validated and measured by qPCR techniques. However, given a small number of biological samples yet a large number of genes, this problem suffers from the difficulty of high computational complexity and imposes challenges to the accuracy of identification statistically.

**Results:**

In this paper, we propose a parsimonious model representation and develop efficient algorithms for identification. Particularly, we derive an equivalent model subject to a sum-to-zero constraint in penalized linear regression, where the correspondence between nonzero coefficients in these models is established. Most importantly, it reduces the model complexity of the traditional approach from the quadratic order to the linear order in the number of candidate genes, while overcoming the difficulty of model nonidentifiablity. Computationally, we develop an algorithm using the alternating direction method of multipliers (ADMM) to deal with the constraint. Numerically, we demonstrate that the proposed method outperforms the traditional method in terms of the statistical accuracy. Moreover, we demonstrate that our ADMM algorithm is more computationally efficient than a coordinate descent algorithm with a local search. Finally, we illustrate the proposed method on a prostate cancer dataset to identify gene pairs that are associated with pre-operative prostate-specific antigen.

**Conclusion:**

Our findings demonstrate the feasibility and utility of using gene pairs as biomarkers.

## Background

In biomedical research, gene identification has been critical towards understanding and predictive modeling, whose activities are associated with biological phenotype, disease status, or clinical outcome. These genes, referred to as biomarkers, are further utilized for predictive modeling to facilitate scientific investigation, clinical diagnosis, prognosis, and treatment developments. In this discovery process, the expression levels of candidate genes are measured through genomic techniques enabling thousands of genes simultaneously, permitting monitoring molecular variation on a genome-wide scale [1] and providing more precise and reliable diagnosis [2]. As widely used techniques, DNA microarray, parallel qPCR, and RNA-Seq measure gene expression at the mRNA level. Yet, two major issues emerge with regard to the utilization of gene expression. First, the number of genes greatly exceeds that of biological samples typically, with tens of thousands of genes in the presence of up to only a few hundred biological samples or observations. As a result, inference tends to be unstable, misleading, or even invalid due to high statistical uncertainty, in addition to extremely high cost of computation. This, in turn, demands reliable and accurate methods of identification. Second, prior to any analysis, raw gene expressions must be normalized to compensate for differences in labeling, sample preparation, and detection methods. A common practice focuses on normalization of each sample’s raw expression based on remaining ones in the same dataset, known as between-sample normalization, often in the forms of sample-wise scaling in RNA-Seq data [3]. However, such a normalization requires recomputation when a new sample is removed or added from the dataset, imposing computational challenges for large studies. Moreover, any analysis using selected genes based on one dataset may be sensitive to normalization, leading to non-generalizable and/or non-reproducible scientific findings [4].

To address the foregoing challenges, a modeling method based on gene pairs was first presented in the top-scoring pair (TSP) classifier by [5] and later implemented by [6]. Compared to predictors based on individual genes, gene pair-based predictors are more robust to normalization and have better predicting or classifying accuracy. Another advantage of gene-pair based predictive modeling is its ease of evaluation and validation by qPCR methods. Ideally, to use qPCR to measure a single gene’s expression level, one applies the delta-Ct method [7], in which the differenced Ct values between the gene of interest and another housekeeping gene such as GAPDH measures gene expression level. However, between sample variation of a housekeeping gene may be large, imposing a great challenge [8]. In this sense, gene-pair based modeling removes the requirement of housekeeping genes since the differenced Ct values between the two genes of interest can be directly treated as a measurement. Consequently, the two genes in a gene pair serve as internal controls for each other. Due to all these advantages, gene pair-based predictors have been adopted in several cancer studies [9–11].

Despite the advantage of the gene pair approach, due to the combinatorial complexity, identifying the best gene pair, or best combinations of several gene pairs, is statistically and computationally challenging, from all the possible pairs from a pool of tens of thousands of genes. For instance, the TSP algorithm employs a direct search, whose running time grows quadratically in terms of the number of candidate genes. Although in practice one can first identify differentially expressed genes and then perform a restrictive search to these individual genes, such a two-step approach is no longer invariant to normalization and may miss informative pairs in which at most one gene is differentially expressed [5]. The computational problem is even more severe when more than one gene pair is sought, such as in *k*-TSP which involves exactly *k* top disjoint pairs in prediction [12].

Moreover, even though rank-based gene pair predictors like the TSP are robust to normalization, their utility in modeling complex data remains limited. One possible extension is to use ratios of gene expression levels as predictors and use regression models to select gene pairs. In recent years, regression models with penalties enforcing sparsity (such as the Lasso [13], SCAD [14], and TLP [15] penalties) have been widely used for variable selection, and many efficient algorithms have been proposed for fitting such models. One may employ such an approach by treating ratios of gene expression levels from all possible gene pairs as candidate predictors. However, this amounts to a quadratic complexity in the number of candidate genes.

In this paper, we develop a new regression approach and an efficient algorithm for identifying gene pairs associated with biological phenotype or clinical outcome. we propose an equivalent model subject to a sum-to-zero constraint on regression coefficients, where the correspondence between nonzero coefficients in these models is established. The model of this type has been proposed for compositional data [16] and recently for reference point insensitive data [17]. One salient aspect is that this model is more parsimonious, involving only predictors linearly in the number of candidate genes. To deal with the constraint, we develop an efficient algorithm based on the alternating direction method of multipliers (ADMM) [18, 19], for identification and model parameter estimation. The new approach shares not only the benefit of simplicity in interpretation but also a linear complexity. Most importantly, the proposed method substantially improves the statistical accuracy and computation efficiency. Finally, in simulations, the method compares favorably against the traditional method in terms of the accuracy of identification, and our ADMM algorithm is more computationally efficient than a coordinate descent with local search (CD+LS) algorithm of [17].

## Methods

### High-dimensional linear regression

This section proposes predictive models based on combinations of ratios of gene expression levels on the ground that ratios of gene expression levels not only are robust to normalization but also can be easily validated and measured by qPCR techniques.

Given *p* predictors (*x*_1_,…,*x*_*p*_) measuring the expression levels of *p* genes (*g*_1_,…,*g*_*p*_), we consider informative second-order interactions defined by pairwise ratios {*x*_*j*_/*x*_*k*_,1≤*j*<*k*≤*p*} of (*x*_1_,…,*x*_*p*_) with respect to a continuous response such as the pre-operative prostate-specific antigen level measured from prostate cancer patients, as demonstrated in the “Results” section. It is assumed that there are only a small number (i.e., much smaller than *p*) of informative genes. Now consider a regression model in which response *Y* depends on a predictor vector ***x*** in a linear fashion:

1 $$\begin{array}{@{}rcl@{}} Y = f(\boldsymbol{z})+ \epsilon \equiv \boldsymbol{\alpha}^{T} \boldsymbol{z} + \epsilon; \quad \epsilon \sim N(0,\sigma^{2}); \quad \end{array} $$

where ***α***=(*α*_12_,*α*_13_,…,*α*_*p*−1*p*_)^*T*^ and ***z***=(log(*x*_1_/*x*_2_), log(*x*_1_/*x*_3_), …, log(*x*_*p*−1_/*x*_*p*_))^*T*^ are ${q=\!\frac {p(p-1)}{2}}$-dimensional vectors of regression coefficients and predictors, and *ε* is random error that is independent of ***z***. For convenience, for *i*<*j*, we let *α*_*ji*_=−*α*_*ij*_. In Eq. (1), primary reasons for the logarithm of the pairwise ratios {*x*_*j*_/*x*_*k*_,1≤*j*<*k*≤*p*} are two-fold. First, it stabilizes the variance of gene expression levels so that Eq. (1) is suitable. In fact, the logarithm transformation is widely used in the literature on gene expression modeling [20]. Second, it facilitates an efficient model fitting algorithm to be introduced subsequently. Our objective is to identify nonzero coefficients of ***α*** corresponding to informative gene pairs based on gene expression.

There are several challenges for identification of informative ratios within the framework of Eq. (1), in which *p* may greatly exceed the sample size *n*, known as high-dimensional regression. Normally, one may apply a feature selection method such as the Lasso [13] for this task. Unfortunately, however, high-dimensionality of Eq. (1) impedes the accuracy of feature selection in the presence of noise in addition to computational cost, which are roughly proportional to *p* ^2^. To overcome these difficulties, we propose an alternative yet equivalent model of Eq. (1) through a more parsimonious representation involving one linear constraint.

The next proposition says that *f*(***z***) in Eq. (1) has an equivalent representation with only *p*-variables. In a sense, it achieves the objective of dimensionality reduction.

#### **Proposition 1**

The following equivalent form of *f*(***z***) in Eq. (1) is as follows:

2 $$\begin{array}{@{}rcl@{}} f(\boldsymbol{z})= \sum_{j=1}^{p} \beta_{j} \log x_{j}, \quad \beta_{j}=\sum_{k\neq j}\alpha_{jk}. \end{array} $$

Importantly, $\sum _{j=1}^{p} \beta _{j}=0$.

Based on Proposition 1, we derive an equivalent model of Eq. (1):

3 $$\begin{array}{@{}rcl@{}} Y = \boldsymbol{\beta}^{T} \tilde{\boldsymbol{x}} + \epsilon, \quad \sum_{j=1}^{p} \beta_{j}=0, \quad \epsilon \sim N(0,\sigma^{2}); \end{array} $$

where $\tilde {\boldsymbol {x}}=(\log x_{1},\ldots,\log x_{p})$ and ***β***=(*β*_1_,…,*β*_*p*_)^*T*^. Most critically, the correspondence between coefficients of ***α*** and ***β*** is established by Eq. (2), where Eq. (1) and Eq. (3) can have different number of nonzero coefficients, which is because of the reparametrization and the sum-to-zero constraint. For instance, suppose that *α*_12_=3, *α*_23_=−2, and *α*_*ij*_=0 otherwise in Eq. (1), then *β*_1_=3, *β*_2_=−5, *β*_3_=2, and *β*_*j*_=0 otherwise in Eq. (3). Model Eq. (3) has been proposed for compositional data [16] and recently also for reference point insensitive data [17]. Here [16] established model selection consistency and bounds for the resulting estimator.

In contrast to Eq. (1), Eq. (3) contains only *p* instead of $\frac {p(p-1)}{2}$ predictors, subject to the sum-to-zero constraint for the regression coefficients. In other words, model Eq. (3) is more parsimonious than model Eq. (1) in terms of the number of active parameters in a model. As a result, there can not be a one-to-one correspondence between ***α*** and ***β***. It is shown in Eq. (2) that the value of ***β*** in Eq. (3) is uniquely determined by that of ***α*** in Eq. (1). The inverse does not hold – many values of ***α*** in Eq. (1) correspond to the same value of ***β*** in Eq. (3). The non-existence of one-to-one correspondence between ***α*** and ***β*** is due to the fact that model Eq. (1) is largely non-identifiable. In fact, for any cycle formed by sequence *i*_1_,*i*_2_,…,*i*_*k*_,*i*_*k*+1_=*i*_1_, we can add any constant *c* to the $\alpha _{i_{j}i_{j+1}}$’s formed by adjacent indices without changing the model. That is, we can construct ***α*** ^′^ where $\alpha ^{\prime }_{i_{j}i_{j+1}}=\alpha _{i_{j}i_{j+1}}+c$ for *j*=1,…,*k* and $\alpha ^{\prime }_{ij}=\alpha _{ij}$ otherwise and model Eq. (1) with ***α*** is equivalent to that with ***α*** ^′^. Therefore, when we obtain a solution ***β*** by solving Eq. (3), due to the argument above, there are an infinite number of ***α*** that are related to ***β*** through Eq. (2). Among them, we would like to choose the “simplest” one. In this paper, we define the “simplest” ***α*** to be the one(s) with the minimum *L*_1_ norm, where the *L*_1_-norm of a vector ***y***=(*y*_1_,…,*y*_*p*_) is $||\boldsymbol {y}||_{1}=\sum _{i=1}^{p}|y_{i}|$. In practice, given an estimate of ***β*** from (3), an estimate of ***α*** can be obtained using Algorithm 1 below.

#### **Algorithm 1**

(Peeling) Given an estimate of $\boldsymbol {\beta }, \hat {\boldsymbol {\beta }}=\left (\hat {\beta }_{1},\cdots, {\hat {\beta }}_{p}\right)^{T}$ satisfying the sum-to-zero constraint $\sum _{j=1}^{p}\hat \beta _{j}=0$, initialize $\tilde {\boldsymbol {\beta }}$ as $\hat {\boldsymbol {\beta }}$ and $\hat {\boldsymbol {\alpha }}$ as ${\hat {\alpha }}_{j,k}=0$ for all 1≤*j*<*k*≤*p*.

**Step 1:** Identify one positive and one negative $\tilde {\beta }_{j}$’s, say $\tilde {\beta }_{k_{1}}>0$ and $\tilde {\beta }_{k_{2}}<0$, where *k*_1_ and *k*_2_ are two distinct indices from {1,⋯,*p*}. For instance, $\tilde {\beta }_{k_{1}}$ and $\tilde {\beta }_{k_{2}}$ can be taken as the most positive and most negative (ties can be broken arbitrarily) $\tilde {\beta }_{j}$’s. This can always proceed as long as not all $\tilde {\beta }_{j}$’s are zero.

**Step 2:** Set $\hat \alpha _{k_{1} k_{2}}=\min \left (|\tilde {\beta }_{k_{1}}|, |\tilde {\beta }_{k_{2}}|\right)$. For instance, $\tilde {\beta }_{1} = 1.5$ and $\tilde {\beta }_{2}=-0.5$, then set $\hat {\alpha }_{12}=0.5$.

**Step 3:** Subtract $\hat {\alpha }_{k_{1} k_{2}}$ from $\tilde {\beta }_{k_{1}}$ and $-\hat {\alpha }_{k_{1} k_{2}}$ from $\tilde {\beta }_{k_{2}}$ to make one of them zero, that is, $\tilde {\beta }_{k_{1}}\leftarrow \tilde {\beta }_{k_{1}}-\hat {\alpha }_{k_{1} k_{2}}$ and $\tilde {\beta }_{k_{2}} \leftarrow \tilde {\beta }_{k_{2}}+\hat {\alpha }_{k_{1} k_{2}}$. In the previous example, $\tilde {\beta }_{1} \leftarrow 1$ and $\tilde {\beta }_{2} \leftarrow 0$.

**Step 4:** Repeat Steps 1–3 until all components of $\tilde {\boldsymbol {\beta }}$ become zero.

Algorithm 1 terminates in at most *p* steps because the number of nonzeros in $\tilde {\boldsymbol {\beta }}$ decreases by either 1 or 2 after each iteration. Note that $\tilde {\beta }_{k_{1}}$ and $\tilde {\beta }_{k_{2}}$ identified in Step 1 may not be unique. Therefore it may lead to different $\hat {\boldsymbol {\alpha }}$’s. Importantly, this algorithm always yields a minimum *L*_1_-norm ***α*** estimate (see Proposition 5 later in this section).

The following two propositions characterize properties of such ***α*** with respect to its representations.

#### **Proposition 2**

(Minimum *L*_1_-norm of ***α***)

Given ***α*** and ***β*** satisfying Eq. (2), the following conditions are equivalent:

(A) For all ***α*** ^′^ satisfying (2), ||***α***||_1_≤||***α*** ^′^||_1_.
(B) For all 1≤*i*,*j*,*k*≤*p*, *i*≠*j*, *j*≠*k*, *α*_*ij*_ *α*_*jk*_≤0.
(C) $||\boldsymbol {\alpha }||_{1}=\frac 12||\boldsymbol {\beta }||_{1}$.

#### **Proposition 3**

Given ***α*** and ***β*** satisfying Eq. (2), the following conditions are equivalent:

(D) For all ***α*** ^′^≠***α*** satisfying Eq. (2), ||***α***||_1_<||***α*** ^′^||_1_.
(E) The conditions in Proposition 2 are met by ***α***. Furthermore, there does not exist distinct (*j*_1_,*k*_1_,*j*_2_,*k*_2_) such that $\alpha _{j_{1}k_{1}}\neq 0$ and $\alpha _{j_{2}k_{2}}\neq 0$ simultaneously.
(F) There exists *j* such that $|\beta _{j}|=\sum _{i\neq j}|\beta _{i}|$. Correspondingly, *α*_*ij*_=*β*_*i*_ for all *i*≠*j* and *α*_*ik*_=0 for all *i*≠*j*, *k*≠*j*.

The following proposition establishes the relations between the numbers of nonzero elements of ***α*** and ***β*** under different settings.

#### **Proposition 4**

Assume that ***α*** and ***β*** satisfy Eq. (2). Let $A=||\boldsymbol {\alpha }||_{0}=\sum _{1 \leq j < k \leq p} I(|\alpha _{jk}| \neq 0)$ and $B=||\boldsymbol {\beta }||_{0}=\sum _{j=1}^{p} I(|\beta _{j}| \neq 0)$ denote the numbers of nonzero elements of ***α*** and ***β***, respectively. Then,

(G) *B*≤2*A*.
(H) If ***α*** and ***β*** satisfy the conditions in Proposition 2, then $2\sqrt {A}\leq B\leq 2A$.
(I) If ***α*** and ***β*** satisfy the conditions in Proposition 3 and ***α***≠***0*** and ***β***≠***0***, then *B*=*A*+1.

In view of condition (H) in Proposition 4, if those conditions of Proposition 2 are met with *B*=2 or 3, then those of Proposition 3 must be satisfied.

#### **Proposition 5**

For any $\hat {\boldsymbol {\beta }}$ satisfying the sum-to-zero constraint, the corresponding $\hat {\boldsymbol {\alpha }}$ produced by Algorithm 1 satisfies Proposition 2.

The proofs of the propositions are supplied in Appendix.

### Constrained penalized likelihood

Given model Eq. (3), a random sample of *n* observations $(\tilde {\boldsymbol {x}}_{i}, Y_{i})_{i=1}^{n}$ is obtained, based on which the log-likelihood function *l*(***β***) can be written as $l(\boldsymbol {\beta })=\frac {-1}{2 \sigma ^{2}} \sum _{i=1}^{n} \left (Y_{i}-{\boldsymbol {\beta }}^{T} \tilde {\boldsymbol {x}}_{i}\right)^{2}$. In a high-dimensional situation, model Eq. (3) is overparameterized when *p*>*n*, and hence that *l*(***β***) has multiple maximizers. Towards this end, we introduce a constrained penalized likelihood as a generalization of the Lasso regression using *L*_1_-regularization.

4 $$\begin{array}{@{}rcl@{}}  \text{min} -l(\boldsymbol{\beta}) + \lambda \sum_{j=1}^{p} |\beta_{j}|, \quad \text{subject to} \sum_{j=1}^{p} \beta_{j}=0. \end{array} $$

Minimization of Eq. (4) in ***β*** yields its minimizer $\hat {\boldsymbol {\beta }}$. Since the term *σ* ^2^ can be absorbed into the regularization coefficient *λ* in the penalized likelihood, we set *σ*=1 in the objective function for simplicity.

In contrast to the Lasso problem, Eq. (4) has one additional linear constraint. The coordinate descent algorithm has been shown to be very efficient for solving *L*_1_-penalized problems [21] since the nondifferentiable *L*_1_ penalty is separable with respect to *β*_*j*_’s. However, the sum-to-zero constraint destroys the separability so that the coordinate descent algorithm can no longer guarantee convergence. In [17], the authors proposed adding additional diagonal moves and random local search to the coordinate descent algorithm, which improves the chance for convergence.

To deal with this convex optimization subject to linear constraints, we develop an algorithm using the alternating direction method of multipliers (ADMM) [18, 19] to solve iteratively, see Appendix for details. In each iteration, we derive an analytic updating formula to expedite convergence of ADMM, and convergence is guaranteed by a result in Section 3.1 of [18]. We compare our ADMM algorithm with the algorithm proposed in [17] in the “Results” section.

## Results

### Comparison of ADMM and CD+LS algorithms

This section compares our ADMM algorithm with a coordinate descent with local search (CD+LS) algorithm of [17] for Eq. (4) with respect to computation efficiency through one simulated example. The CD+LS algorithm is implemented in R package zeroSum (version 1.0.4, https://github.com/rehbergT/zeroSum). In this example, we consider correlated predictors, that is, $\tilde {\boldsymbol {x}}_{i}$’s are drawn iid from *N*(0,***V***) and are independent of *ε*_*i*_’s that are sampled from *N*(0,1), and ***V*** is a *p*×*p* matrix whose *ij*th element is 0.5^|*i*−*j*|^. Moreover, the true *β*_*j*_’s are drawn iid from *N*(0,1) and then centered to have a sum zero, and *λ* is fixed as 1. Then, their rates for successful convergence and running times are recorded for the ADMM and the CD+LS algorithms over 20 simulations. Particularly, a precision or tolerance error of 10^−10^ is used for both algorithms. Successful convergence is reported if a solution from an algorithm satisfies the sum-to-zero constraint with a tolerance error of 10^−8^, and both the solution and its objective value are no further than 10^−8^ to the optimal solution and its corresponding objective value in terms of the *L*_2_-distance. Here, the optimal solution is defined as the one, among the two solutions from the two algorithms, having the minimal objective value and satisfying the sum-to-zero constraint.

Four different settings are compared, ranging from low- to high-dimensional situations. As showed in Table 1, the proposed ADMM algorithm outperforms the CD+LS algorithm with respect to both convergence guarantee and running time.

**Table 1:** Comparison of ADMM and CD+LS algorithms

		% Success Rate		Time (s)	
*p*	*n*	ADMM	CD+LS	ADMM	CD+LS
20	50	100.0	100.0	0.03	0.0
		(0.0)	(0.0)	(0.01)	(0.0)
100	100	100.0	100.0	0.06	1.3
		(0.0)	(0.0)	(0.00)	(0.1)
100	500	100.0	80.0	0.14	0.6
		(0.0)	(9.2)	(0.04)	(0.2)
200	1000	100.0	80.0	0.54	3.9
		(0.0)	(9.2)	(0.12)	(0.4)

Sample means (standard errors in parentheses) of rates for successful convergence (in percentages), and running times (in seconds), based on 20 simulation replications, for the proposed ADMM and CD+LS algorithms

### Comparison of the proposed method and the Lasso

This section examines effectiveness of the proposed method through simulated examples. Specifically, the proposed method is compared with the Lasso in terms of predictive accuracy and identification of the true model, where the Lasso is implemented in R package glmnet [21].

Simulated examples are generated with correlation structures as to be analyzed. These simulations are designed to examine various operating characteristics of the proposed method with respect to (*p*,*n*), noise level *σ* ^2^, and correlation structures among predictors in Eqs. (1) and (3). For tuning, *λ* is searched over 100 grid points that are uniformly spaced (in the log-scale) between 10^4^ and 10^−2^. An independent testing dataset with 1000 randomly generated data points are used to find the optimal *λ* which minimizes the mean squared error (MSE).

For performance metrics, an independent validation dataset with 1000 randomly generated data points are used to evaluate the performance of the fitted model in terms of mean squared error (MSE) and *R* ^2^. To assess robustness of the approaches under data normalization, we randomly add sample-wise shifts from *N*(0,1) to the validation dataset. Furthermore, we consider other two metrics for parameter estimation and the quality of identification of zero elements of true ***α*** in Eq. (1) and ***β*** in Eq. (3). For parameter estimation, we use the relative error (RE) for estimating the true regression coefficients $\boldsymbol {\gamma }^{0}=\left (\gamma ^{0}_{1},\ldots,\gamma ^{0}_{p}\right)^{T}$, defined as

$$\begin{array}{@{}rcl@{}} \text{RE} =\frac{||\tilde{\boldsymbol{\gamma}} - {\boldsymbol{\gamma}}^{0}||_{2}} {||\boldsymbol{\gamma}^{0}||_{2}}, \end{array} $$

where $\tilde {\boldsymbol {\gamma }}=(\tilde {\gamma }_{1},\ldots,\tilde {\gamma }_{p})^{T}$ are estimated regression coefficients. This metric allows for accounting for different scales between ***α*** and ***β***. For accuracy of identification, we use the false identification rate (FR), defined as

$$\begin{array}{@{}rcl@{}} \text{FR} = 1-\frac{|\tilde\Gamma|\cap|\Gamma^{0}|}{|\Gamma^{0}|}, \end{array} $$

where $\Gamma ^{0}=\{j | \gamma ^{0}_{j}\neq 0\}$ and $\tilde \Gamma =\{j | \tilde \gamma ^{\prime }_{j}\neq 0\}$, with $\tilde \gamma ^{\prime }$ being a truncated version of $\tilde \gamma $ such that only the coefficients with the |*Γ* ^0^| largest absolute values are retained, and all others are zeroed out. Our simulated example concerns correlation structures among predictors. In Eqs. (1) and (3), log***x***_*i*_’s are iid from *N*(0,***V***) and are independent of *ε*_*i*_’s that are iid from *N*(0,*σ* ^2^), and ***V*** is a *p*×*p* matrix whose *ij*th element is 0.5^|*i*−*j*|^, ***α***=(*α*_12_,*α*_13_,…,*α*_*p*−1*p*_)^*T*^. Three settings for ***α*** are considered:

1. *α*_12_=1, *α*_13_=0.5, *α*_24_=0.5, and *α*_*jk*_=0 otherwise, which does not satisfy the conditions defined in Proposition 2 with *β*_1_=1.5, *β*_2_=*β*_3_=*β*_4_=−0.5, and *β*_*j*_=0 for *j*≥5.
2. *α*_12_=1, *α*_13_=0.5, *α*_24_=−0.5, and *α*_*jk*_=0 otherwise, which satisfies the conditions defined in Proposition 2 but does not satisfy the conditions defined in Proposition 3 with *β*_1_=1.5, *β*_2_=−1.5, *β*_3_=−0.5, *β*_4_=0.5, and *β*_*j*_=0 for *j*≥5.
3. *α*_12_=1, *α*_13_=0.5, *α*_14_=0.5, and *α*_*jk*_=0 otherwise, which satisfies the conditions defined in Proposition 3 with *β*_1_=2, *β*_2_=−1, *β*_3_=*β*_4_=−0.5, and *β*_*j*_=0 for *j*≥5.

The proposed method is compared with the Lasso in two models, corresponding to the gene-pair level design matrix ***z*** in Eq. (1) and $\tilde {\boldsymbol {x}}$ in Eq. (3) (without the sum-to-zero constraint), referred to as Lasso 1 and Lasso 2, respectively. These three methods are examined on simple and difficult situations correspondingly with (*p*,*n*,*σ*)=(25,50,0.5),(100,25,0.2). Then the values of MSE, *R* ^2^, RE, and *FR* are reported. As suggested in Table 2, the proposed method performs better or the same compared with Lasso 1 and Lasso 2 in terms of accuracy and robustness across all the six situations. The improved performance is attributed to a reduced number of candidate parameters in Eq. (1) than Eq. (3), as well as to the sum-to-zero constraint introduced in Eq. (3). Interestingly, the false identification rates of the proposed method are almost zero in three low-noise setting of (*p*,*n*,*σ*)=(20,50,0.5) regardless if the conditions in Propositions 2 and 3 are met, and are small in the other three settings. In contrast, Lasso 1 has a higher relative error and false identification rate. While Lasso 2 has similar relative error and false identification rate as the proposed method, it has higher MSE and lower *R* ^2^ in all settings, due to its non-robustness to sample-wise scaling without the sum-to-zero constraint. Overall, all three methods perform better in the low-noise situation of (*p*,*n*,*σ*)=(20,50,0.5) than the high-noise situation of (*p*,*n*,*σ*)=(100,25,0.2). Across the three settings of ***α***, the performance of the proposed method is rather stable. However, Lasso 1 performs much worse for settings in which ***α*** fails to satisfy the conditions in Proposition 3, corresponding to non-uniqueness of the representation of ***α***. Most critically, when ***α*** satisfies the conditions in Proposition 3, the proposed method continues to outperform its counterpart in terms of the performance metrics. Overall, the proposed method achieves our objective.

**Table 2:** Comparison of the proposed method and the Lasso in simulations

Setting	(*p*,*n*,*σ*)	Method	MSE	*R* ^2^	RE	FR
1	(20, 50, 0.5)	Lasso 1	0.29	0.89	0.77	0.33
			(0.01)	(0.00)	(0.01)	(0.00)
		Lasso 2	0.31	0.88	0.14	0.00
			(0.01)	(0.00)	(0.01)	(0.00)
		Proposed	0.29	0.89	0.13	0.00
			(0.01)	(0.00)	(0.01)	(0.00)
1	(100, 25, 0.2)	Lasso 1	0.19	0.92	0.80	0.37
			(0.07)	(0.03)	(0.02)	(0.02)
		Lasso 2	0.24	0.89	0.22	0.06
			(0.08)	(0.03)	(0.03)	(0.03)
		Proposed	0.19	0.92	0.21	0.04
			(0.07)	(0.03)	(0.03)	(0.03)
2	(20, 50, 0.5)	Lasso 1	0.32	0.89	0.44	0.20
			(0.01)	(0.00)	(0.02)	(0.04)
		Lasso 2	0.36	0.88	0.14	0.00
			(0.02)	(0.01)	(0.01)	(0.00)
		Proposed	0.32	0.89	0.14	0.01
			(0.01)	(0.00)	(0.01)	(0.01)
2	(100, 25, 0.2)	Lasso 1	0.42	0.84	0.52	0.37
			(0.07)	(0.02)	(0.03)	(0.06)
		Lasso 2	0.63	0.76	0.32	0.22
			(0.09)	(0.03)	(0.03)	(0.05)
		Proposed	0.41	0.85	0.31	0.24
			(0.07)	(0.02)	(0.03)	(0.05)
3	(20, 50, 0.5)	Lasso 1	0.30	0.93	0.18	0.00
			(0.01)	(0.00)	(0.02)	(0.00)
		Lasso 2	0.33	0.92	0.11	0.00
			(0.01)	(0.00)	(0.01)	(0.00)
		Proposed	0.30	0.93	0.11	0.00
			(0.01)	(0.00)	(0.01)	(0.00)
3	(100, 25, 0.2)	Lasso 1	0.15	0.96	0.25	0.00
			(0.02)	(0.01)	(0.03)	(0.00)
		Lasso 2	0.26	0.93	0.16	0.01
			(0.08)	(0.02)	(0.02)	(0.01)
		Proposed	0.15	0.96	0.15	0.00
			(0.02)	(0.01)	(0.01)	(0.00)

Sample means (standard errors in parentheses) of mean squared error (MSE), *R* ^2^, relative error (RE) and false identification rate (FR), based on 20 simulation replications, for the proposed method and the Lasso

### An application to a real RNA-Seq dataset

This section applies the proposed method to a prostate adenocarcinoma (PRAD) RNA-Seq dataset published as part of The Cancer Genome Atlas (TCGA) project [22]. Particularly, we identify gene pairs that are associated with pre-operative prostate-specific antigen (PSA), an important risk factor for prostate cancer. Towards this end, we download normalized gene expression data from the TCGA data portal (https://tcga-data.nci.nih.gov/docs/publications/tcga/). As described by TCGA, tissue samples from 333 PRAD patients were sequenced using the Illumina sequencing instruments. While raw sequencing reads were processed and analyzed using the SeqWare Pipeline 0.7.0 and MapspliceRSEM workflow 0.7 developed by the University of North Carolina, read alignment was performed using MapSplice [23] to the human reference genome, and gene expression levels were estimated using RSEM [24] with gene annotation GAF 2.1, and further normalized so that the upper quartile count is 1,000 in each sample. All these steps were performed by the TCGA consortium.

In our experiment, the normalized RSEM gene expression estimates are used, excluding samples with missing pre-operative PSA values and genes for which the average normalized expression level is lower than 10. This prepossessing step yields *p*=15,382 genes and *n*=187 samples. Furthermore, we run Pearson correlation tests for each gene between log-transformed expression levels and log-transformed pre-operative PSA levels, and exclude genes with false discovery rate (FDR) values (calculated using the Benjamini-Hochberg [25] method based on the *p*-values from the Pearson correlation tests) larger than 0.01. Consequently, only 520 genes are retained in the analysis, on which we fit model Eq. (3) using the proposed ADMM algorithm.

**Fig. 1:**
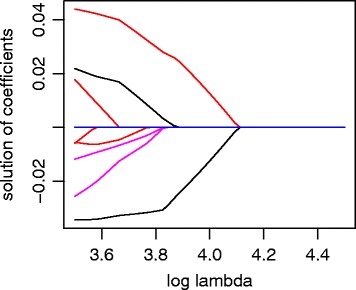
Solution paths of the model fitting with *p*=520 genes

To visualize the selection result, we display the solution paths of the model fitting. As shown in Fig. 1, the first pair of genes entering the model are PTPRR and KRT15. While PTPRR is a member of the protein tyrosine phosphatase (PTP) family, which is known to be related with prostate cancer [26, 27], KRT15 is a member of the keratin gene family, which is known to be associated with breast cancer [28] and lung cancer [29]. Interestingly, we find no publication record on PubMed with keywords such as “KRT15 AND PSA” or “KRT15 AND prostate”. By correlating log expression levels and log PSA levels in the 187 patients, we find that both PTPRR and KRT15 are significantly correlated with PSA levels (*r*=0.28 and *p*<10^−4^ for PTPRR, *r*=−0.33 and *p*<10^−5^ for KRT15). Not surprisingly, their log-ratio is even more strongly correlated with log PSA levels (*r*=0.41 and *p*<10^−8^), demonstrating the potential of using gene pairs as biomarkers.

The other selected genes are HIST1H1E, LRAT, LCN2, KCNN4, RHOU, and EPHA5 in the order of them entering the model, among which LRAT [30], LCN2 [31], RHOU [32], and EPHA5 [33] are known to link to prostate cancer, and HIST1H1E and KCNN4 are connected to myeloma [34] and pancreatic cancer [35], respectively.

To demonstrate the scalability of our proposed method, we employ the proposed ADMM algorithm to fit Eq. (3) with all the *p*=15,382 genes without pre-screening. In this situation, the first pair of genes entering the model are BCL8 and KRT15, where BCL8 is known to be associated with lymphoma [36]. The other selected genes are PTPRR, LRAT and LCN2, which are very similar to the foregoing results based on pre-screening. By comparison, fitting the corresponding model Eq. (1) using a standard Lasso algorithm, such as glmnet [21], would be practically prohibitive, which requires storing a design matrix of $\frac {p(p-1)}{2}\times n \approx 22\times 10^{9}$ elements. To further demonstrate robustness of the proposed method with respect to data normalization, we randomly scale the gene expression levels, both along the gene dimension and the sample dimension, mimicking the gene length normalization and the library size or sequencing depth normalization, respectively. Numerically, we find that the solution path fitted using the randomly scaled data is always identical to that fitted using the original data.

## Discussion

Model Eq. (3) has been proposed for compositional data [16] and recently for reference point insensitive data [17]. In this article, we explore Eq. (3) for the identification of gene pairs as biomarkers, enjoying robustness to sample-wise scaling normalization (which is a common practice for RNA-Seq data) and simplicity of validation and measurement by qPCR techniques. Through Propositions 1–4, we establish the relationship between models Eq. (1) and Eq. (3). Additionally, we develop an efficient ADMM algorithm for solving model Eq. (3), which is guaranteed to converge and is shown to be highly competitive in terms of computational efficiency.

One interesting yet important issue of model Eq. (3) is determination of the value of ***α***. One proposal is choosing the ***α*** to minimize the *L*_0_-norm instead of *L*_1_-norm. However, in such a situation, it remains unclear what kind of conditions as those in Propositions 2, 3 and 4 may be. Furthermore, minimization of the *L*_0_-norm in ***α*** continues to be challenging by itself due to non-convexity and discontinuity of the *L*_0_-function. Therefore, our approach based on the *L*_1_-norm gives rise to convex minimization, which is easier to manage.

One important aspect of model Eq. (3) is that it enables to identify gene pairs in an unbiased manner without any prior knowledge of the known biology of the disease. However, in some situations, information regarding the disease of interest is available from previous studies. Then the prior knowledge needs to be integrated for gene pair identification so that more probable subset of genes or pathways should have a higher chance of being selected. This can be accomplished through weighted regularization with weights $\{\lambda _{k}\}_{k=1}^{p}$, with a large weight corresponding to a small chance of being selected. Moreover, in some other situations, gene pairs are constrained in that gene pairs are formed only between relevant genes from the same pathway. This can be achieved by replacing the Lasso penalty by either a (sparse) group Lasso penalty [37] and/or the simple equality constraint by a set of constraints, each corresponding to a given pathway of interest. Finally, some non-convex penalties such as the SCAD penalty [14] and the TLP penalty [15] can be used as opposed to the Lasso penalty to achieve a higher accuracy of selection at an expense of computation.

For a large-scale problem, an ADMM algorithm may have a linear convergence rate. To expedite convergence, an inexact ADMM algorithm may be useful in our setting, which has been shown recently to lead to a substantial improvement over the standard ADMM algorithm [19]. Furthermore, parallelization of our ADMM algorithm may achieve further scalability, which is one advantage of ADMM algorithms over many other optimization techniques [18].

One extension of Eq. (1) is generalized linear models such as logistic regression or other predictive models like support vector machine. In such a situation, the proposed method for Eq. (1) is directly applicable to gene pair identification with some modifications. Further investigation is necessary.

## Conclusion

In conclusion, the experimental results demonstrate that gene pairs can be used as robust biomarkers which can tolerate sample-wise scaling normalization. Furthermore, using *L*_1_ penalized regression with equality constraints, the model fitting can be formulated as a convex optimization problem which can be solved efficiently using the proposed ADMM algorithm. This approach has the potential to discover novel and reliable biomarkers for biological or clinical studies.

## Appendix

### Proofs of propositions

**Proof of Proposition 1:** From Eq. (1), we have

$$\begin{array}{@{}rcl@{}} f(\boldsymbol{z})&=&\sum_{j=1}^{p}\sum_{k=j+1}^{p}\alpha_{jk}(\log x_{j} - \log x_{k})\\ &=&\sum_{j=1}^{p}\log x_{j}\left(\sum_{k=j+1}^{p}\alpha_{jk}-\sum_{k=1}^{j-1}\alpha_{kj}\right)\\ &=&\sum_{j=1}^{p}\log x_{j}\sum_{k\neq j}\alpha_{jk}\\ &=&\sum_{j=1}^{p}\beta_{j}\log x_{j}. \end{array} $$

Furthermore

$$\begin{array}{@{}rcl@{}} \sum_{j=1}^{p}\beta_{j} &=& \sum_{j=1}^{p}\sum_{k\neq j}\alpha_{jk}\\ &=&\sum_{j=1}^{p} \sum_{k= j+1}^{p} \alpha_{jk}-\sum_{j=1}^{p} \sum_{k=1}^{j} \alpha_{kj}\\ &=&\sum_{j=1}^{p} \sum_{k= j+1}^{p} \alpha_{jk}-\sum_{k=1}^{p} \sum_{j=k+1}^{p} \alpha_{kj}\\ &=&0. \end{array} $$

This completes the proof.

**Proof of Proposition 2:** We show (A) ⇒(B), (B) ⇒(C) and (C) ⇒(A), respectively.

(A) ⇒(B): We prove by contradiction. Without loss of generality, assume that *α*_12_≥*α*_23_>0. Then, we construct ***α*** ^′^ such that $\alpha ^{\prime }_{12}=\alpha _{12}-\alpha _{23}$, $\alpha ^{\prime }_{23}=0$, $\alpha ^{\prime }_{13}=-\alpha _{13}+\alpha _{23}>0$ and $\alpha ^{\prime }_{ij}=\alpha _{ij}$ otherwise. Easily, ***α*** ^′^ satisfies Eq. (2) and ||***α*** ^′^||_1_−||***α***||_1_=−2*α*_13_<0, which contradicts (A).

(B) ⇒(C): By (B), *α*_*ij*_ and *α*_*jk*_ always have opposite signs. This, together with the definition that *α*_*ji*_=−*α*_*ij*_, *α*_*ji*_ and *α*_*jk*_ always have the same sign, where 0 can be regarded as an arbitrary sign. Therefore, from Eq. (2), we have

$$\begin{array}{@{}rcl@{}} \frac12||\boldsymbol{\beta}||_{1}=\frac12\sum_{j=1}^{p}|\beta_{j}|=\frac12\sum_{j=1}^{p}\left|\sum_{k\neq j}\alpha_{jk}\right|\\ =\frac12\sum_{j=1}^{p}\sum_{k\neq j}|\alpha_{jk}|=||\boldsymbol{\alpha}||_{1}. \end{array} $$

(C) ⇒(A): For any ***α*** ^′^ satisfying Eq. (2), we have

$$\begin{array}{@{}rcl@{}} ||\boldsymbol{\alpha}||_{1}=\frac12||\boldsymbol{\beta}||_{1}=\frac12\sum_{j=1}^{p}|\beta_{j}|=\frac12\sum_{j=1}^{p}\left|\sum_{k\neq j}\alpha^{\prime}_{jk}\right|\\ \leq\frac12\sum_{j=1}^{p}\sum_{k\neq j}|\alpha^{\prime}_{jk}|=||\boldsymbol{\alpha}^{\prime}||_{1}. \end{array} $$

This completes the proof.

**Proof of Proposition 3:** We show (D) ⇒(E), (E) ⇒(F) and (F) ⇒(D), respectively.

(D) ⇒(E): As in the proof of Proposition 2, we assume, without loss of generality, that *α*_24_≥*α*_13_>0. Then, we construct ***α*** ^′^ such that $\alpha ^{\prime }_{13}=0$, $\alpha ^{\prime }_{14}=\alpha _{14}+\alpha _{13}$, $\alpha ^{\prime }_{23}=\alpha _{23}+\alpha _{13}$, $\alpha ^{\prime }_{24}=\alpha _{24}-\alpha _{13}$ and $\alpha ^{\prime }_{ij}=\alpha _{ij}$ otherwise. Easily, ***α*** ^′^ also satisfies Eq. (2), and ||***α*** ^′^||_1_≤||***α***||_1_, which contradicts (D).

(E) ⇒(F): From (E), there exists a *j* such that *α*_*ik*_=0 for all *i*≠*j* and *k*≠*j*. From Eq. (2) and (B) in Proposition 2, $\beta _{i}=\sum _{k\neq i}\alpha _{ik}=\alpha _{ij}, \text {for all}i\neq j$, and $ |\beta _{j}|=\left |\sum _{k\neq j}\alpha _{jk}\right |=\sum _{k\neq j}|\alpha _{jk}|=\sum _{k\neq j}|\beta _{k}|. $

(F) ⇒(D): Suppose that ***α*** ^′^ satisfies Eq. (2) and the conditions in Proposition 2. From (B), we know for all *k*≠*j*, $\alpha ^{\prime }_{jk}$ have the same sign. From Eq. (2), (C) and (F), we have

$${} \frac12||\boldsymbol{\beta}||_{1}=|\beta_{j}|=\left|\sum_{k\neq j}\alpha^{\prime}_{jk}\right|=\sum_{k\neq j}|\alpha^{\prime}_{jk}|\leq||\boldsymbol{\alpha}^{\prime}||_{1}=\frac12||\boldsymbol{\beta}||_{1}$$

Therefore, we must have $\sum _{k\neq j}|\alpha ^{\prime }_{jk}|=||\boldsymbol {\alpha }^{\prime }||_{1}$. That is, $\alpha ^{\prime }_{ik}=0$ for all *i*≠*j*, *k*≠*j*. Furthermore, for any *i*≠*j*, we have $\beta _{i}=\sum _{k\neq i}\alpha ^{\prime }_{ik}=\alpha ^{\prime }_{ij}.$ Therefore, ***α*** ^′^=***α***, implying the uniqueness of ***α***. This completes the proof.

**Proof of Proposition 4:**

(G): By Eq. (2), *β*_*j*_≠0 only if at least for some *k*≠*j*, *α*_*jk*_≠0. Based on ***α*** and ***β***, we can construct an undirected graph *G*=(*V*,*E*) with *p* vertices such that there is an edge between vertices *i* and *j* if and only if *α*_*ij*_≠0. We know that *β*_*j*_ can not be nonzero unless vertex *V*_*j*_ has a degree of at least 1. Since the total number of edges is *A*, we know that $B\leq \sum _{j=1}^{p}I(degree(V_{j})>0)\leq \sum _{j=1}^{p} degree(V_{j})=2A$.

(H): Suppose ***α*** satisfies the conditions defined in Proposition 2. If *α*_*ij*_≠0, let *α*_*ij*_ be the weight associated with edge connecting *V*_*i*_ and *V*_*j*_. By condition (B), for any cycle in the graph formed by sequence *i*_1_,*i*_2_,…,*i*_*k*_,*i*_*k*+1_=*i*_1_, we know that weights associated with adjacent edges (i.e., $\alpha _{i_{j-1}i_{j}}$ and $\alpha _{i_{j}i_{j+1}}$) always have opposite signs. Therefore, the number of edges in the cycle has to be an even number, which means that the graph has to be a bipartite graph. It is then easy to see that $A\leq \left (\frac {B}{2}\right)^{2}$ for such graphs. That is, $2\sqrt {A}\leq B$.

(I): Suppose ***α***≠0 satisfies the conditions defined in Proposition 3. By condition (F), *B*=*A*+1. This completes the proof.

**Proof of Proposition 5:** It suffices to show that $\hat {\alpha }$ satisfies (C) of Proposition 2. Note that $\tilde {\boldsymbol {\beta }}$ in Algorithm 1 satisfies the sum-to-zero constraint at each step of iteration before termination. In the beginning, $\|\hat {\boldsymbol {\alpha }}\|_{1}=0$ and $\|\tilde {\boldsymbol {\beta }}\|_{1}=\|\hat {\boldsymbol {\beta }}\|_{1}$. After each iteration, $\|\hat {\boldsymbol {\alpha }}\|_{1}$ is increased by $\hat \alpha _{k_{1} k_{2}}=\min (|\tilde {\beta }_{k_{1}}|, |\tilde {\beta }_{k_{2}}|)$, and $\|\tilde {\boldsymbol {\beta }}\|_{1}$ is decreased by $2\hat \alpha _{k_{1} k_{2}}$. In the end, $\|\tilde {\boldsymbol {\beta }}\|_{1}=0$, and therefore $\|\hat {\boldsymbol {\alpha }}\|_{1} =\frac {1}{2} \|\hat {\boldsymbol {\beta }}\|_{1}$. This completes the proof.

### ADMM algorithm for solving Eq. (4)

We adopt the notation in [18] and reformulate Eq. (4) as

5 $$  \begin{aligned} &\text{min}\ (1/2)\|\boldsymbol{Ax}-\boldsymbol{b}\|^{2}_{2}+\lambda\|\boldsymbol{z}\|_{1} \\ &\text{subject to}\ \boldsymbol{Cx}=d, \boldsymbol{x}-\boldsymbol{z}=\boldsymbol{0}. \end{aligned}  $$

where ***x*** are the parameters of interest. If ***C***=1^*T*^ and *d*=0, we will have all coefficients sum up to 0. When there is an intercept in the model, we can add a scalar 0 as the first element in ***C***, meaning that we do not have constraint on the intercept. Similarly, as a convention we also do not penalize the intercept. Denoting $\boldsymbol {B}=\left [\begin {array}{ll}\boldsymbol {C}\\\boldsymbol {I} \end {array}\right ]$, $\boldsymbol {D}=\left [\begin {array}{cc}\boldsymbol {0}\\\boldsymbol {-I}\end {array}\right ]$, and $\boldsymbol {d}=\left [\begin {array}{ll}d\\\boldsymbol {0}\end {array}\right ]$, the two equality constraints can be simplified as ***B*** ***x***+***D*** ***z***=***d***. To use the ADMM algorithm [18], we form the augmented Lagrangian

$$\begin{array}{@{}rcl@{}} L_{\rho}(\boldsymbol{x},\boldsymbol{z},\boldsymbol{y})& = & (1/2)\|\boldsymbol{Ax}-\boldsymbol{b}\|^{2}_{2}+\lambda\|\boldsymbol{z}\|_{1} \\ & & +(\rho/2)\|\boldsymbol{B}\boldsymbol{x}+\boldsymbol{D}\boldsymbol{z}-\boldsymbol{d}+\boldsymbol{u}\|^{2}_{2}-(\rho/2)\|\boldsymbol{u}\|^{2}_{2}, \end{array} $$

with $\boldsymbol {u}^{k}=(1/\rho)\boldsymbol {y}^{k}=\left [\begin {array}{ll}u_{1}\\ \boldsymbol {u_{2}}^{p}\end {array}\right ]$ where *u*_1_ is a scalar and $\boldsymbol {u_{2}} \in \mathbb {R}^{p\times 1}$.

Let $\boldsymbol {E}=\left [\begin {array}{cc}\boldsymbol {A}\\ \sqrt {\rho } \boldsymbol {C}\end {array}\right ]$, the ADMM algorithm consists of the following iterations

$${} {{\begin{aligned} \boldsymbol{x}^{k+1}&:=(\boldsymbol{E}^{T}\boldsymbol{E}+\rho \boldsymbol{I})^{-1}\left(\boldsymbol{A}^{T}\boldsymbol{b}+\rho\left(\boldsymbol{z}^{k}-\boldsymbol{C}^{T}u_{1}^{k}+\boldsymbol{C}^{T}d-\boldsymbol{u_{2}}^{k}\right)\right)\\ \boldsymbol{z}^{k+1}&:=S_{\lambda/\rho}\left(\boldsymbol{x}^{k+1}+\boldsymbol{u_{2}}^{k}\right)\\ \boldsymbol{u}^{k+1}&:=\boldsymbol{u}^{k}+\left(\boldsymbol{B}x^{k+1}+\boldsymbol{D}z^{k+1}-\boldsymbol{d}\right). \end{aligned}}} $$

The ***x*** update can be accelerated by caching an initial factorization. Suppose the dimension of ***E*** is *m*×*p*. If *m*<*p*, we cache the factorization of *I*+*ρ* ***E*** ***E*** ^*T*^ (with dimension *m*×*m*) and use the matrix inversion lemma

$$\left(\rho \boldsymbol{I}+\boldsymbol{E}^{T}\boldsymbol{E}\right)^{-1}=\boldsymbol{I}/\rho-\boldsymbol{E}^{T}\left(\boldsymbol{I}+1/\rho \boldsymbol{E}\boldsymbol{E}^{T}\right)^{-1}\boldsymbol{E}/\rho^{2}$$

to update ***x***. Otherwise, we cache the factorization of *ρ* ***I***+***E*** ^*T*^ ***E*** (with dimension *p*×*p*) and use back- and forward- solve to update ***x*** directly. The iteration stops when the primal and dual residuals are smaller than their corresponding tolerances,

$$\|\boldsymbol{r}^{k+1}\|_{2}\leq \epsilon^{pri}\quad \text{and}\ \quad \|\boldsymbol{s}^{k+1}\|_{2} \leq \epsilon^{dual},$$

where

$${} {{\begin{aligned} \boldsymbol{r}^{k+1}&=\boldsymbol{B}\boldsymbol{x}^{k+1}+\boldsymbol{D}\boldsymbol{z}^{k+1}-\boldsymbol{d},\\ \boldsymbol{s}^{k+1}&=\rho\boldsymbol{B}^{T}\boldsymbol{D} \left(\boldsymbol{z}^{k+1}-\boldsymbol{z}^{k}\right),\\ \epsilon^{pri}&=\sqrt{p+1} \epsilon^{abs}+\epsilon^{rel} \text{max}\ \left\{ \| \boldsymbol{B} \boldsymbol{x}^{k+1}\|_{2},\|-\boldsymbol{z}^{k+1}\|_{2}, \|\boldsymbol{d}\|_{2} \right\},\\ \epsilon^{dual}&=\sqrt{p} \epsilon^{abs}+\epsilon^{rel}\|\rho \boldsymbol{B}^{T}\boldsymbol{u}^{k+1}\|_{2}. \end{aligned}}} $$

Usually, the relative stopping criteria is chosen to be *ε* ^*rel*^=10^−4^, and the choice of absolute stopping criteria *ε* ^*abs*^ depends on the scale of the variable values. See Boyd et al. [18] for details. To compute a solution path for a decreasing sequence of *λ* values, we adopt the approach in Friedman et al. [21] and use warm starts for each *λ* value. The sequence of *λ* values are either provided by the user, or we begin with *λ*_*max*_=∥***A*** ^*T*^ ***b***∥_*∞*_ for which all the coefficients are equal to 0. We set *λ*_*min*_=*ε*_*λ*_ *λ*_*max*_, where *ε*_*λ*_ is a small value, such as 0.01, and generate a decreasing sequence of 100 *λ* values from *λ*_*max*_ to *λ*_*min*_ on the log-scale.

## Abbreviations

ADMM: Alternating direction method of multipliers
CD+LS: Coordinate decent with local search
DNA: Deoxyribonucleic acid
FDR: False discovery rate
FR: False identification rate
iid: Independent identically distributed
Lasso: Least absolute shrinkage and selection operator
mRNA: Messenger RNA
MSE: Mean squared error
PRAD: prostate adenocarcinoma
PSA: Prostate-specific antigen
PTP: Protein tyrosine phosphatase
qPCR: Quantitative polymerase chain reaction
RE: Relative error
RNA: Ribonucleic acid
RNA-Seq: RNA sequencing
RSEM: RNA-Seq by expectation-maximization
SCAD: Smoothly clipped absolute deviation
TCGA: The cancer genome atlas
TLP: Truncated *L*_1_ penalty
TSP: Top-scoring pair
